# Unique and differential protein signatures within the mononuclear cells of HIV-1 and HCV mono-infected and co-infected patients

**DOI:** 10.1186/1559-0275-9-11

**Published:** 2012-09-07

**Authors:** Nawal M Boukli, Vivekananda Shetty, Luis Cubano, Martha Ricaurte, Jordana Coelho-dos-Reis, Zacharie Nickens, Punit Shah, Andrew H Talal, Ramila Philip, Pooja Jain

**Affiliations:** 1 Universidad Central del Caribe School of Medicine, Biomedical Proteomics Facility Department of Microbiology and Immunology, Bayamon, Puerto Rico; 2 Immunotope, Inc., Pennsylvania Biotechnology Center, Doylestown, PA, USA; 3 Department of Microbiology and Immunology, and the Drexel Institute for Biotechnology and Virology Research, Drexel University College of Medicine, 3805 Old Easton Road, Doylestown, PA, USA; 4 Center for the Study of Hepatitis C, Weill Cornell Medical College, New York, NY, USA

**Keywords:** HIV-1, HCV, HIV-1/HCV, 2D-GE, Mass spectrometry, Pro- and anti-apoptotic fingerprinting, Proteomics

## Abstract

**Background:**

Pathogenesis of liver damage in patients with HIV and HCV co-infection is complex and multifactorial. Although global awareness regarding HIV-1/HCV co-infection is increasing little is known about the pathophysiology that mediates the rapid progression to hepatic disease in the co-infected individuals.

**Results:**

In this study, we investigated the proteome profiles of peripheral blood mononuclear cells from HIV-1 mono-, HCV mono-, and HIV-1/HCV co-infected patients. The results of high-resolution 2D gel electrophoresis and PD quest software quantitative analysis revealed that several proteins were differentially expressed in HIV-1, HCV, and HIV-1/HCV co-infection. Liquid chromatography-mass spectrometry and Mascot database matching (LC-MS/MS analysis) successfully identified 29 unique and differentially expressed proteins. These included cytoskeletal proteins (tropomyosin, gelsolin, DYPLSL3, DYPLSL4 and profilin-1), chaperones and co-chaperones (HSP90-beta and stress-induced phosphoprotein), metabolic and pre-apoptotic proteins (guanosine triphosphate [GTP]-binding nuclear protein Ran, the detoxifying enzyme glutathione S-transferase (GST) and Rho GDP-dissociation inhibitor (Rho-GDI), proteins involved in cell prosurvival mechanism, and those involved in matrix synthesis (collagen binding protein 2 [CBP2]). The six most significant and relevant proteins were further validated in a group of mono- and co-infected patients (n = 20) at the transcriptional levels.

**Conclusions:**

The specific pro- and anti- apoptotic protein signatures revealed in this study could facilitate the understanding of apoptotic and protective immune-mediated mechanisms underlying HIV-1 and HCV co-infection and their implications on liver disease progression in co-infected patients.

## Background

Hepatitis C virus (HCV) is one of the most common viruses to co-infect individuals with human immunodeficiency virus type 1 (HIV-1). Of the 1 million individuals infected with HIV-1 in the United States, approximately 30%-40% are co-infected with HCV [1]. Globally, the prevalence of HIV-1/HCV co-infection is substantial, with significant overlap in geographical areas and types of populations affected [2]. HIV-1 and HCV share similar routes of transmission; intravenous drug use may be the main cause of the high rates of HIV-1/HCV co-infection (up to 90%) [2]. In addition, many individuals who received multiple blood transfusions prior to mandatory HIV-1 and HCV screening have been found to be co-infected [1]. As antiretroviral therapy became more effective, HCV infection emerged as an important cause of morbidity and mortality in HIV-infected individuals. In co-infected individuals, as the incidence of chronic and vertical HCV infection increases, the rates of HCV-mediated end-stage liver disease [3] as well as cirrhosis, liver failure, and hepatocellular carcinoma (HCC) [2], also increase. Although global awareness regarding HIV-1/HCV co-infection is increasing and extended therapeutic programs are becoming available for this population, little is known about the pathophysiology that mediates the rapid progression to hepatic disease in the co-infected individuals [4].

Liver disease caused by HIV-1/HCV co-infection is characterized by inflammation and cell death. Recently it has been reported that the HIV and HCV envelope proteins may induce apoptosis and inflammation in hepatocytes via a novel pathway involving collaborative signaling [5]. Increasing evidence emphasizes that comparative proteomics analysis of differentially expressed proteins associated with HIV/AIDS could potentially result in identification of biomarkers and drug targets [6-8]. While a few proteomics studies have focused on the analysis of serum samples from HIV-1, HCV mono- and co-infected patients, the proteomic analysis of peripheral blood mononuclear cells (PBMCs) has seldom been reported for these patients, which are highly relevant in the context of HIV-1/HCV co-infection. The main cell targets for HIV are mononuclear leukocytes bearing CD4 and the chemokine receptors CCR5 and CXCR4 [9-13]. While HCV primarily infects and replicates in hepatocytes, there is accumulating evidence about extrahepatic replication of HCV, especially in PBMCs. The highly conserved 5’ untranslated region (UTR) of HCV RNA has been shown to vary between PBMCs and plasma of the same person [14,15]. In addition, a compartmental distribution of HCV quasispecies and genotypes has been demonstrated [15,16] suggesting that HCV alters its replication inside non-hepatic cells. Lymphoid cells may act as HCV reservoirs that could help it establish chronic infection. Different studies have shown the presence of low levels of HCV RNA in serum and lymphoid cells after spontaneous or Interferon/Ribavirin-induced resolution of chronic hepatitis C [17,18]. The occult HCV persistence in lymphoid cells may pose a big challenge to the clinical management of HCV infection because the current techniques to detect HCV RNA in lymphoid cells or serum are not very sensitive. Moreover, in patients with sustained virological response (SVR), small quantities of HCV RNA might persist in liver or PBMC for up to 9 years [18]. It was also demonstrated that HCV might persist and replicate in the liver and PBMC of healthy, anti-HCV antibody-positive, serum HCV RNA-negative patients who have persistently normal ALT levels [19]. This could present a potential risk for transmission and reactivation especially in the presence of HIV. In fact, the presence of HCV RNA at the end of treatment correlated with relapse among HIV/HCV co-infected patients [20]. Interestingly, HCV RNA was shown to re-emerge in apparent SVRs receiving immune suppressive therapy [21,22]. This proved that HCV still persists even years after Interferon/Ribavirin-induced resolution from plasma and continued immune surveillance is required to prevent its recurrence even in SVRs [21]. Therefore, it is critical to understand the influence of both these viruses on peripheral lymphoid cells.

A few screening studies have used known serum markers identified in HCV infected patients to predict the progression of liver disease in co-infected patients [23-26]. In addition, some studies have shown that there is correlation between elevated serum concentrations of α-fetoprotein (AFP) and the occurrence of HCC, which may provide a useful surrogate marker for disease [27]. However, AFP levels fluctuate in chronically infected individuals and AFP is a poor biomarker for small tumors [28]. Differential proteomic analysis and proteomic fingerprinting of HIV-1 infected macrophages have been shown to predict HIV-1-associated cognitive impairment [29,30]. Although these models have generally performed well with regard to the differentiation of fibrosis, it remains critical to identify novel biomarkers that will enable us not only to differentiate between degrees of liver fibrosis but also to elucidate the molecular mechanisms of HIV-1- and HCV-mediated pathogenesis.

In the present study, we investigated the comprehensive proteome profiling of HIV-1 mono-, HCV mono-, and HIV-1/HCV co-infected patients in comparison with the uninfected control samples. Specifically, we assessed the effects of these two chronic viral infections, alone and in combination, on the PBMCs using a comparative proteomic analysis in an attempt to identify differentially expressed proteins in terms of the mechanisms underlying HIV-1 and HCV mono- and co-infection. Herein, we report that HIV-1, HCV, and their co-infection differentially and uniquely express key proteins involved in viral survival, replication and inflammatory responses signifying the molecular mechanisms in agreement with previous clinical observations. The identified changes further highlight protein signatures possibly involved in pro-inflammatory response and immune suppression that are highly implicated in progression of liver disease in chronic HCV infection, which could potentially be used as drug targets.

## Materials and methods

### Ethics statement

All the procedures involving the human PBMCs were approved by Universidad del Caribe (Puerto Rico) and Weill Cornell Medical College (USA) Ethics committees and written informed consent was provided by study participants prior to blood collection.

### Clinical samples

PBMCs were obtained from the age- and gender-matched clinical samples including normal control individuals (HIV-1^-^/HCV^-^) and individuals with HIV-1 mono-infection (HIV-1^+^/HCV^-^), HCV mono-infection (HIV-1^-^/HCV^+^), and HIV-1/HCV co-infection (HIV-1^+^/HCV^+^). Total mononuclear cell population was prepared using Ficoll-Paque Plus density gradient centrifugation as per manufacturer’s instructions (Amersham Biosciences, Uppsala, Sweden). The clinical information on the patients utilized in the study is given in Table 1. Initially, one sample from each group was analyzed by triplicate 2D gels followed by LC/MS/MS analyses of the selected spots. The key observations made from these samples were confirmed in the extended number of samples as listed in Table 1.

**Table 1:** Patients utilized in the study

**Patients code**	**Sex**	**Infection status**	**CD4**	**HIV load**	**HCV load**
H1*	**F**	**HIV**	**288**	**140222**	**NUL**
H2	M	HIV	56	373429	NUL
H3	F	HIV	400	46189	NUL
H4	M	HIV	218	95726	NUL
H5	M	HIV	508	1665	NUL
HC1*	**F**	**HIV/HCV**	**325**	**<75**	**79000**
HC2	M	HIV/HCV	422	<74	63999
HC3	M	HIV/HCV	590	<75	216000
HC4	F	HIV/HCV	399	<75	1200000
HC5	F	HIV/HCV	331	123	5,070,000
C1*	**F**	**HCV**	**517**	**NUL**	**85426**
C2	F	HCV	397	NUL	>1000000
C3	**F**	HCV	354	NUL	>1000000
C4	M	HCV	516	NUL	417070
C5	M	HCV	389	NUL	>1000000
N1*	**F**	**Negative**	**N/A**	**NUL**	**NUL**
N2	F	Negative	N/A	NUL	NUL
N3	M	Negative	N/A	NUL	NUL
N4	M	Negative	N/A	NUL	NUL
N5	M	Negative	N/A	NUL	NUL

* Samples utilized for 2D-gel elctrophoresis N/A = not available.

### *Protein extraction*

For each 100 mg of PBMC pellet, 1 mL of Mammalian Protein Extraction Reagent (Pierce Protein Research Products, Thermo Fisher Scientific, Rockford, IL) was added and centrifuged at 14,000 rpm for 15 minutes. The supernatant was transferred to a new tube with equal volume of the sample buffer containing 7 M urea, 2 M thiourea, 0.5 M Tris–HCl, pH 8.5, 4% CHAPS (3-[(3-cholamidopropyl)-dimethylammonio]-1-propanesulfonate), 65 mM dithiothreitol, 0.5% IPG (immobilized pH gradient) buffer, 0.5 M ethylenediamine tetra-acetic acid, and 1 mM protease inhibitor cocktail. The mixture was centrifuged at 3,500 rpm at 12°C for 1 h with centrifugal filter devices (Centricon, 3-kDa cutoff) as per manufacturer’s instructions (Amicon; Millipore, Bedford, MA). This process was repeated 4 times to remove salts and lipids. After desalting, the concentration of proteins was measured using the modified Bradford assay.

### *High resolution two dimensional gel electrophoresis*

One hundred and fifty micrograms of protein from cell lysates was solubilized for 30 min with 2D rehydration/ sample buffer (7 M urea, 2 M thiourea, 1% ASB-14, 40 mM Tris) and 2% Bio-Rad IPG buffer, pH 3–10. Bio-Rad 11 cm ReadyStrip pH 3–10NL IPG strips were used to separate proteins according to charge. Solubilized proteins were adsorbed into the gel strip overnight and were then focused according to their isoelectric point with the Bio-Rad Protean IEF System. The program used was the following: 250 V rapid voltage ramping for 30 min, 10,000 V slow voltage ramping for 60 min, and 10,000 V rapid voltage ramping for 50 kV hours. DryStrips were first rehydrated in 186 μL of rehydration buffer and mixed with 150 μg of HIV, HCV and HIV/HCV co-infected PBMCs and PBMC control proteins separately. The strips were then loaded onto 6-18% SDS-PAGE gels on 13.3 × 8.7 cm and run at 50 V overnight to complete the second dimension of protein separation. ReadyPrep Overlay Agarose was added on top of the strip to secure it and included bromophenol blue tracking dye. A molecular standard was used to estimate relative mass (Mr). Gels were pre-rinsed with water, and stained overnight with Bio-safe coomassie stain as stated in manufacturers protocol. Gels were destained in water, and scanned with the Versadoc Model 1000 system (Bio-Rad, CA). Gel image analyses were performed with PD Quest software (Bio-Rad) version 7.4.0. Individual spot volumes for each gel were normalized relative to the total spot volume of that gel. Normalized spot volume data from each experimental set were analyzed the Student’s t-test (P < 0.05 was regarded as significant). Spots ≥2-fold higher/lower were considered to be differentially regulated. To analyze reproducibility of our results, this procedure was repeated three times with each sample.

### *Image analysis and protein quantification*

The 2D gel imaging and analysis software PDQuest (Bio-Rad) version 7.4.0 was used for gel-to-gel matching and for identifying differences between control and treated samples. Each of the four samples (control, HIV-1^+^, HCV^+^, and HIV-1^+^/HCV^+^) was represented by three independent biological replicates of 2D gels, giving a total of 12 analyzed gels. The gel images were normalized for staining intensities using the PDQuest software. Each matched protein spot was assigned a unique sample spot protein number. Selected spots from individual gel images were first matched to at least two other replicate gels using the “classic gel match” algorithm function. Refined maps for individual gels were created through use of land-marking and manual matching. Once a master gel was created for each sample, higher analysis set matching was performed to identify qualitative changes. Quantitative changes were determined by normalization followed by matching triplicate samples from individual time points to other time points. For each analysis, the 2D gel from the control PBMC sample was set as the standard. Based on these settings, analysis sets for each of the individual infected sample cohorts were created for a representative gel using the normalized parameters to identify qualitative changes and a 2-fold increase/decrease to identify quantitative changes. Subsequently, proteins that consistently displayed a 2-fold or greater increase or decrease in protein expression over three separate experiments, in each of the four cohorts, were validated and reported. A minimum 2-fold change was considered for up-regulated proteins and 0.7-fold for down-regulated proteins.

### *In-gel digestion of proteins*

In-gel digestion of proteins was carried out by following the RapiGest SF protocol (Waters Corp., Milford, MA). Briefly, the excised protein spots were washed with water and cut into smaller pieces. These gel pieces were de-stained using 50% acetonitrile (ACN) in water and dried by vacuum centrifugation. Thereafter, 30 μL of 50 mM ammonium bicarbonate (AB) containing 0.1% of RapiGest SF was added to the dried gel pieces and proteins were reduced with dithiothreitol (100 μM in 100 mM AB) at 65°C for 45 min. Dithiothreitol excess was removed and cysteines were alkylated with iodoacetamide (55 μm in 100 μM AB) by incubating the reaction mixture in the dark for 30 min. After alkylation, excess buffer containing iodoacetamide was removed and gel pieces were dried under vacuum. The alkylated proteins in dried gel pieces were then digested by trypsin (Promega, Madison, WI) (5 ng/μL in 50 mM AB) overnight at 37°C in a water bath. The tryptic peptides were extracted in three steps: first by adding 100% ACN, second with a solution of 0.1% formic acid in water, and finally with 100% ACN (including sonication for 5 min in each step). All three peptide extractions were pooled and RapiGest was removed as per manufacturer’s instructions. The tryptic peptide solutions were dried under vacuum and re-suspended in 0.1% formic acid solution for the LC/MS/MS analysis.

*LC/MS/MS analysis.* A 3000-nano ultimate high-performance liquid chromatography (HPLC) system (Dionex, Sunnyvale, CA) was coupled with an LTQ ion-trap mass spectrometer (Thermo Electron, San Jose, CA) equipped with advanced nanospray source to analyze tryptic peptides obtained from each 2D gel protein spot. Tryptic peptides were injected into the LC-MS/MS system to sequence peptides. As a part of an online sample clean-up, the peptides were first concentrated in a C18 RP trap column (Dionex) and then separated using a 75-μm ID × 15-cm C18 RP analytical column (Dionex) equilibrated in 4% ACN/0.1% FA at a flow rate of 250 nL/min. Mobile phase A was 2% ACN and 0.1% FA in water, and mobile phase B was 0.1% FA and 90% ACN in water. Peptides were separated with a gradient of 4%-50% B in 60 min and 50%-80% in 90 min and eluted directly into an ion-trap mass spectrometer. The mass range in MS mode was 350–1800 Da and in MS/MS mode it was set as 100–2000 Da. The instrument method was set to acquire fragment ion (MS/MS) spectra on the four most abundant precursor ions from each MS scan with a repeat count set of 1 and a repeat duration of 30 seconds. Dynamic exclusion was enabled for 180 seconds.

### *Protein identification*

The proteins were identified by searching the raw tandem MS data in the International Protein Index (IPI) human database with a Sequest search algorithm and by using the Proteome Discoverer 1.1 and Bioworks 3.3.1 software programs (Thermo Electron, San Jose, CA). The database search parameters were as follows: database - IPI human, mass-type monoisotopic precursor and fragment; enzyme - trypsin, threshold - 100, peptide tolerance - 1.5 Da, fragment ion tolerance - 1.0. Modifications: M - oxidation, C - carbamidomethylation, N/Q - deamidation. The search results were filtered with cross-correlation (Xcorr) via charge states (+1: 1.5, +2: 2.0, +3: 2.5, +4: 3.0), delta correlation (ΔCn > 0.1), molecular weight (m/z 500 Da–m/z 2000 Da), and peptide mass accuracy (Δ1.5 Da) parameters. The criteria involved in the selection of proteins in each gel spot were the comparison of experimental pI, molecular weight, and sequence coverage of proteins with that of theoretical pI and molecular weight of proteins detected on 2D gels. However, proteins identified with a single peptide were also selected in view of their high XCorr numbers and high-quality MS/MS spectra with b and y series ions. All the tandem MS data were further verified manually to identify proteins with high confidence.

### *PCR analyses*

Total RNA was extracted from frozen PBMC samples using TRI reagent (Sigma Aldrich, St. Louis, MO) and purified using the RNeasy Mini Kit (Qiagen, Valencia, CA) as previously described [31]. Purified RNAs were transcribed to cDNAs using Omniscript Reverse Transcriptase (4U, Qiagen, Valencia, CA) in the presence of dNTP mix (0.5 mM each dNTP; Qiagen, Valencia, CA) oligo dT15 primer (1 μM, Promega, Madison, WI) and SUPERaseIn RNase Inhibitor (10U, Ambion, Austin, TX) for 1 h at 37°C. Real Time PCR was carried out to evaluate mRNA expression of *Gelsolin* (F/R: ACGGCTGAAGGACAAGAAGA/ TTCCAACCCAGACAAAGACC), *HSP90-beta* (F/R: CGCATGAAGGAGACACAGAA/TCC CATCAAATTCCTTGAGC), *Profilin-1* (F/R: CATCGTGGGCTACAAGGACT/TCCATGCT AAATTCCCCATC), *Rho-GDI* (F/R: GAGCCTGCGAAAGTACAAGG/TCCTTCAGCACAA ACGACTG), *CBP2* (F/R: TGTTCTTCAAGCCACACTGG/CGATTTGCAGCTTTTCCTTC) and *STIP-1* (F/R: GGCAGTATGGATGAGGAGGA/AGCTCCTTGGCTTTGTCGTA), and *GAPDH* (F/R: CAATGACCCCTTCATTGACC/TTGATTTTGGAGGGATCTCG). Real-time PCR was performed on an ABI Prism 7500 Sequence Detector (Applied Biosystems, Foster City, CA). The reaction was run in duplicates and consisted of 25 μl SYBR Green PCR Master Mix (Applied Biosystems), 0.2 μM of each primer, and 2 μl of cDNA in a total volume of 30 μl. The cycling conditions comprised an initial step at 95°C for 10 min followed by 40 cycles at 95°C for 15 sec and annealing and extension at 60°C for 1 min. Dissociation curve analysis was implemented to ensure the presence of a single peak at the correct melting temperature. The C_t_ values for each of the target genes evaluated were normalized with GAPDH C_t_ values for each sample. Fold changes in mRNA expression for the genes were expressed as 2^−ΔCt^.

### *Statistical analysis*

For the image analysis and protein quantification, student *t* test was performed with 95% significance level to determine which proteins were differentially expressed between the infected and control samples. Analyses of significant statistical differences in the mRNA expression obtained for each patient from the four groups tested were evaluated using ANOVA one way and unpaired student *t* test. Values of p < 0.05 were considered as statistically significant. Fold changes were also calculated to express the differences in protein expression between the infected and control samples.

## Results

### *HIV-1, HCV, and HIV-1/HCV co-infected PBMC proteome*

**Figure 1:**
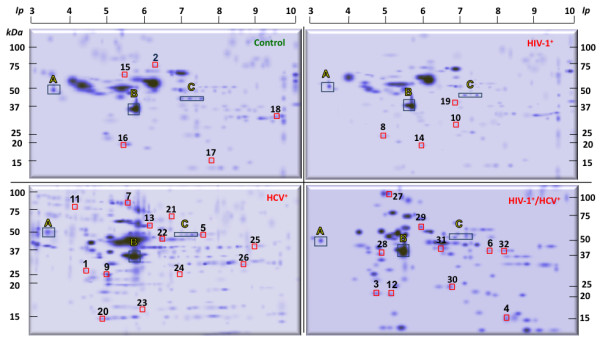
**Color reproduction on the Web.** Overview of two-dimensional gel electrophoresis (2D-GE) showing protein profiles in control, HIV-1^+^, HCV^+^ and HIV-1^+^/HCV^+^ peripheral blood mononuclear cells (PBMCs). The red squares in control samples represent the downregulated proteins while in infected samples represent the up-regulated proteins. The consistently identified proteins as common spots on the 2D gels among all the four conditions are indicated with capital letters. All experiments were performed in triplicate. Representative 2D gel (from which spots were selected) images are provided here.

**Figure 2:**
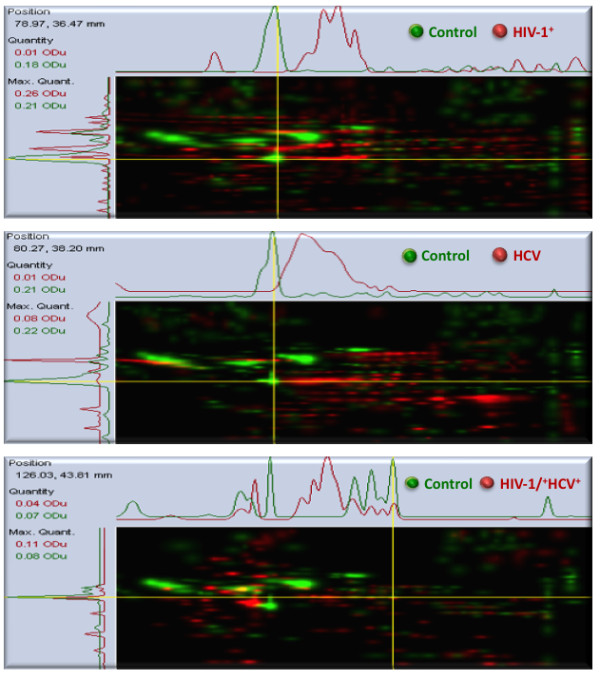
**Color reproduction on the Web and in print.** Multichannel viewer highlighting the overlay of HIV-1+, HCV+, and HIV-1+/HCV + samples versus PBMC control. The images depict a computer-generated color-coded comparison between the three samples and exhibit intensity variations in x- and y- directions.

To facilitate quantitative detection and to maximize identification of changes in the proteome induced by HIV-1, HCV, and HIV-1/HCV PBMCs, cell lysates were subjected to a 2D gel electrophoresis coupled with LC-MS/MS mass spectrometry analysis. Because it was not feasible to sequence all resolved proteins, the selection criteria for sequencing were based on the quantitative analysis of the 2D gel image (Figure 1) with the goal of obtaining a broad scope of the proteins regulated by HIV-1 and HCV alone and in combination. A total of 271 protein spots were detected by gel image analyses of the control samples while 184, 224, and 145 protein spots were detected in HIV-1^+^, HCV^+^, and HIV-1^+^/HCV^+^ PBMCs, respectively (Figure 1). Of these, 153, 177, and 91 proteins, respectively, were increased in expression after HIV-1, HCV, and HIV-1/HCV infections, as measured via Biosafe Coomassie blue staining and the multichannel viewer analyses (Figure 2). Conversely, 31, 47, and 54 spots in the HIV-1, HCV, and HIV-1/HCV samples, respectively, were identified as having decreased expression relative to control. Figure 2 (panel 3) clearly demonstrates a shift in protein expression pattern of the co-infected sample as compared to either of the mono-infected sample.

For protein identification by LC-MS/MS analysis, 4, 10, and 8 differentially expressed spots in HIV-1, HCV, and HIV-1/HCV, respectively (Additional file 1: Table S1), and 1, 7, and 5 uniquely expressed spots in HIV-1, HCV, and HIV-1/HCV, respectively (Additional file 2: Table S2), were selected (significance at *p < 0.05*). These spots were selected from six categories: 1) HIV-1-induced up-regulation; 2) HIV-1-induced down-regulation; 3) HCV-induced up-regulation; 4) HCV-induced down-regulation; 5) HIV-1/HCV-induced up-regulation); and 6) HIV-1/HCV-induced down-regulation. Some proteins were uniquely expressed: four in control, one in HIV, seven in HCV, and five in HIV/HCV. LC-MS/MS experiments and database matching result in successful identification of 29 differentially and uniquely expressed proteins (Additional file 1: Table S1 and Additional file 2: Table S2). These proteins belonged to five major functional categories such as protein synthesis, cytoskeletal proteins, metabolic/glycosylation proteins, and enzymes. The additional data pertinent to the identification of these proteins by mass spectrometry are provided in the Additional file 3: Table S3 and Additional file 3: Table S4.

### *Proteins regulated by HIV-1 infection*

**Figure 3:**
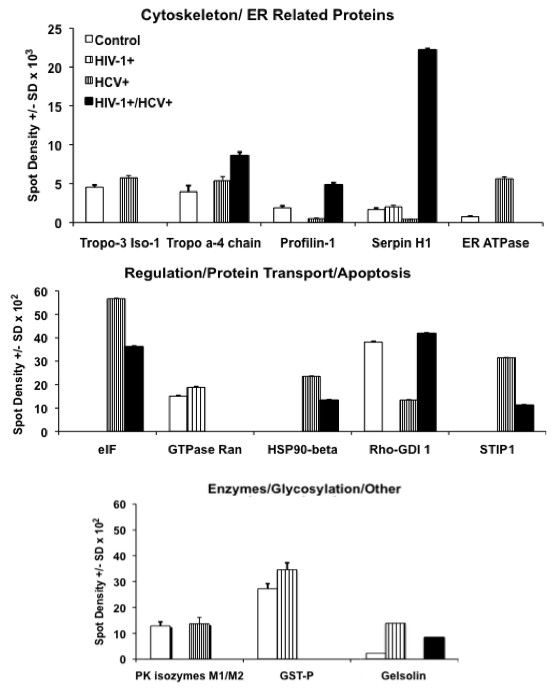
**Comparative spot density values of differentially expressed proteins among control, HIV-1**^**+**^**, HCV**^**+**^**mono-infected, and HIV-1**^**+**^**/HCV**^**+**^**co-infected samples.** Data represent the normalized spot volume (+/− standard deviation) representing the expression level of each protein for the 3 independent experiments in the three conditions versus the three independent experiments in the control.

The cytoskeletal protein gelsolin (spot 2), the molecules involved in protein synthesis, collagen binding protein 2 (CBP2) (spot 6), the detoxification enzyme glutathione S-transferase P (GST) (spot 8), and the protein GTP-binding nuclear protein Ran (GTPase Ran) (spot 10) involved in cell progression and microtubule organization were up-regulated by 6.2-, 2.1-, 2.38-, and 2.2-fold, respectively, as determined by quantifying the spot densities over three replicates in the HIV-1 group as compared with control (*p < 0.05*; n = 3) (Figure 3). We also observed a uniquely expressed spot for HIV-1 corresponding to a protein highly similar to serum albumin (spot 19). HIV-1 infection also led to a decrease in the expression of three cytoskeletal proteins including tropomyosin (spots 1 and 3) and profilin (spot 4) as well as one metabolic protein Rho-GDP dissociation inhibitor (Rho-GDI) (spot 12). The down-regulation of Rho-GDI could be an HIV-1 pro-survival mechanism since it is an activator of nicotinamide adenine dinucleotide phosphate (NADPH), which results in the formation of superoxide [32]. The remainder of the down-regulation changes identified occurred in two proteins, the glycolytic protein pyruvate kinase (spot 5) and the ER ATPase (spot 7).

### *Proteins regulated by HCV infection*

Among the proteins differentially expressed by HCV were the cytoskeletal proteins tropomyosin alpha-4 isoform 1 (spot 1) and tropomyosin alpha-4 (spot 3), which were up-regulated by 2.27- and 2.36-fold, respectively (Figure 3). We also identified an ER ATPase (spot 7) that was up-regulated by 7.85-fold (Figure 3). Molecules involved in protein synthesis such as eukaryotic translation initiation, factor 3 subunit K (EIF3K; spot 9), HSP90-beta (spot 11), and the stress-induced phosphoprotein (spot 13) were present in the HCV PBMCs but were not detected in the control samples. We also observed uniquely expressed spots for HCV corresponding to a protein similar to prostaglandin E synthase 3 (spot 20), DPYSL3 (dihydropyrimidinase-like 3) (spot 21), DPYSL4 (spot 22), nucleoside diphosphate kinase A (spot 23), thioredoxin (spot 24), a protein highly similar to CBP2 (spot 25), and malate dehydrogenase, mitochondrial (MDH2; spot 26) as located in the 2D gel and identified by LC/MS/MS analysis. DPYSL3 and DPYSL4 are involved in semaphorin signaling and subsequent rearrangement of the cytoskeleton. One protein needs special mention; the protein highly similar to CBP2, which was specifically increased in HCV infected PBMCs in agreement with previous findings showing that HCV promotes expression of collagen [33].

**Figure 4:**
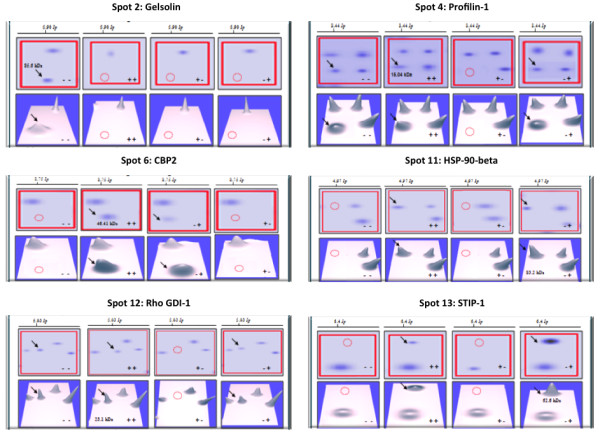
**Normalized mRNA levels of *Gelsolin*, *HSP-90beta*, *Profilin-1*, *Rho-GDI-1*, *CBP2 *and *STIP-1 *obtained by Real-Time PCR.** The results were plotted as discrete plots that display individual normalized mRNA levels, represented by the symbols for control (squares), HIV-1^+^ (triangles), HCV^+^ (lozenges) and HIV-1^+^/HCV^+^ (dots) groups. The black dashes represent the median calculated for each group. The data represents one of the two independent experiments performed in duplicate for each experiment. Statistical significance is demonstrated by *p < 0.05* or *p < 0.01*. Non-significant *p* values were represented as n.s.

Furthermore, we observed that HCV infection led to a decrease in the expression of the cytoskeletal protein profilin (spot 4), which was down-regulated by 3.91-fold. We also demonstrated that HCV infection induced a decreased PBMC expression of the cell synthesis of CBP2 (spot 6), which was down-regulated by 4.05-fold; the metabolic protein GST (spot 8); the cell growth protein GTPase Ran (spot 10); and Rho-GDI, which was down-regulated by 2.84-fold (Figure 4). Similar to HIV-1, the down-regulation of Rho-GDI may represent an HCV pro-survival mechanism as well by reducing the activation of NADPH.

### *Proteins regulated by HIV-1/HCV co-infection*

**Figure 5:**
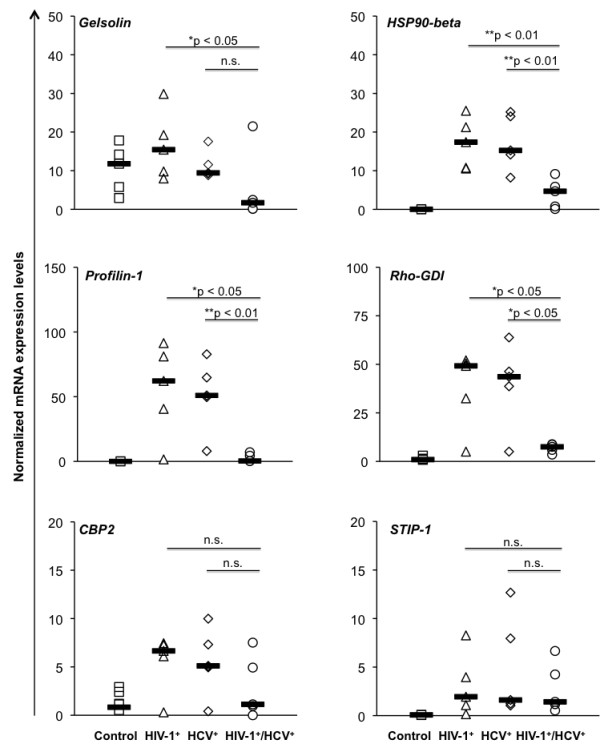
**2D-GE and 3D view of 6 selected differentially expressed proteins identified by Tandem Mass Spectrometry Detection (LC-MS/MS).** The gels are representing control, HIV-1, HCV and HIV-1/HCV samples from left to right.

In HIV-1/HCV co-infected sample, the cytoskeletal proteins gelsolin (spot 2), tropomyosin alpha-4 chain (spot 3), and profilin-1 (spot 4) were up-regulated by 3.7-, 2.17- and 2.54-fold, respectively (Figure 4). CBP2 (spot 6) was up-regulated by 13.28-fold as compared with the control. Molecules involved in protein synthesis such as EIF3K (spot 9), HSP90-beta (spot 11), and the stress-induced phosphoprotein (spot 13) were present in the HIV-1/HCV co-infected PBMCs but were not detected in either the control or HIV-1 infected PBMCs. The fact that spots 9, 11, and 13 were specifically up-regulated in HCV and - HIV-1/HCV infected PBMCs suggests that these could be related to HCV infection and not to HIV-1 infection, since they were not expressed in the HIV-1 infected PBMC samples. Rho-GDI was up-regulated by 4.2 fold in HIV-1/HCV infected PBMCs as compared with the control. The co-infection of HIV-1/HCV led to the down-regulation of cytoskeletal protein tropomyosin alpha-4 isoform 1 (spot 1), the glycolytic protein pyruvate kinase isozymes M1/M2 (spot 5), an ER ATPase (spot 7), the anti-apoptosis and apoptosis regulator enzyme GST (spot 8), EIF3K (spot 9–2.57-fold), the cell growth protein GTPase Ran (spot 10), the chaperone HSP90-beta (spot 11–2.77-fold), and the stress-induced phosphoprotein 1 (STIP-1) (spot 13–2.82-fold). Since, tropomyosin alpha-4 isoform 1, pyruvate kinase isozymes M1/M2, ER ATPase, and STIP-1 were down-regulated in HIV-1 and HIV-1/HCV infection but not in HCV-infected PBMC, their down-regulation is likely related to the presence of HIV-1 infection. Conversely, GST and GTPase Ran were down-regulated in HCV and HIV-1/HCV infection but not in HIV-1 infection, their down-regulation is likely to be related to the presence of HCV infection. The representative 2D-GE, 3-dimensional view, and histogram of six most significant and relevant proteins are given in Figure 5. These proteins were further validated at the mRNA levels in an extended group of patients as listed in Table 1.

### ***Validating Proteomics results by Real-Time PCR***

Real-Time PCR was performed to evaluate mRNA levels of *Gelsolin, HSP90-beta, Profilin-1, Rho-GDI, CBP2 and STIP-1* and results were expressed as normalized Ct values relative to the housekeeping gene (*GAPDH)* for control, HIV-1^+^, HCV^+^ and HIV-1^+^/HCV^+^ samples (Figure 4). Five patients were evaluated in each group for the six genes tested. The data demonstrates the significant (*p < 0.05*) up-regulation of *HSP90-beta, Profilin-1, Rho-GDI, and STIP-1* in all infected samples as compared to control samples. For CBP2, a significant (*p < 0.05*) up-regulation was observed in the mono-infected samples but not in the co-infected samples and for *Gelsolin* no significant difference was observed as compared to control. While comparing mono-infected samples with those of the co-infected samples, statistically significant differences were observed for *Gelsolin* between HIV-1 versus HIV-1/HCV and for *HSP90-beta*, *Profilin-1 and Rho-GDI* between HIV-1 and HCV versus HIV-1/HCV positive samples. Although *CBP2 and STIP-1* demonstrated higher expression levels in the mono-infected samples but the differences were not statistically significant (Figure 4).

Additional analysis comparing paired protein and mRNA levels obtained from the sample employed in both the Proteomics and Real Time PCR analyses showed a similar correlative trend for Gelsolin, HSP90-beta, CBP2 and STIP-1 but not for Profilin-1 and Rho-GDI (data not shown). Profilin-1 and Rho-GDI showed up-regulation of both mRNA and protein in the co-infected samples; however, down-regulation of protein with up-regulation of mRNA was observed in mono-infected samples. This could be due to the fact that increase in mRNA levels does not always correlate with the protein expression since mRNAs could be degraded during the process of translation. The viral-induced post-transcriptional interference is also possible since the discrepancy in mRNA versus protein expression was only observed in the infected samples.

## Discussion

**Figure 6:**
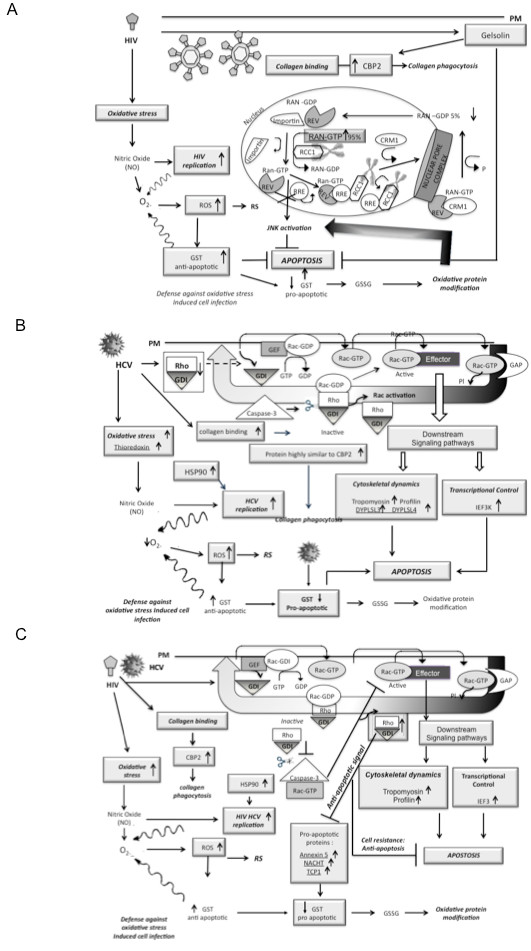
**Color reproduction on the Web.** Proposed model highlighting specific protein signatures triggered by HIV-1 (**A**), HCV (**B**) and HIV-1/HCV (**C**) in peripheral blood mononuclear cells. ROS, reactive oxygen species; RS, reactive substances; GST, Glutathione S-transferase. Arrows up indicate up-regulated proteins and arrows down indicate down-regulated proteins. Underlined proteins are proteins unique to each of the conditions (HIV-1, HCV and HIV-1/HCV samples).

We obtained 2D profiles of the protein expression in PBMCs of HIV-1/HCV mono- and co-infected patients and uninfected controls. Proteins related to protein synthesis, the ER, oxidative stress, the cytoskeleton, metabolism, and glycolysis are differentially and uniquely expressed in PBMCs of HIV-1/HCV mono- and co-infected patients. In general, several up-regulated proteins showed overlap between the HCV mono-infected and HIV-1/HCV co-infected samples, and only two up-regulated proteins, gelsolin and CBP2, were shared by HIV-1 mono-infected and HIV-1/HCV co-infected patient samples. In contrast to the results observed in control cells, a high level expression of CBP2 occurred in infected samples in agreement with previous studies showing that HIV and HCV promote expression of collagen [33,34]. An anti-apoptotic signature through the up-regulation of GST, gelsolin and Ran was detected on HIV-infected cells. The down-regulation of Rho-GDI (known to lead to an apoptotic response) was observed on HCV infections. Gelsolin and Rho-GDI, key proteins that promote cell prosurvival mechanism, were up-regulated in co-infected PBMCs. These identified proteins have diverse cellular functions that will require further in-depth studies to understand the protective and apoptotic immune-mediated mechanisms triggered by HIV-1 and HCV. Figure 6 encompass the HIV-1, HCV and HIV-1/HCV induced differentially expressed proteins detected in this study. In the model, we propose that HIV and HIV-1/HCV induce a pro-survival anti-apoptotic mechanism, to protect the cell from the pathogenic interaction “cross-talk” of the two viruses, while HCV seem to trigger a more apoptotic protein signature.

***HIV up-regulated, CBP2, GST, Ran and Gelsolin.*** CBP2 (also known as HSP47) is an ER resident protein involved in collagen synthesis. The observed up-regulation of CBP2 correlates with previous studies demonstrating significant increases in collagen deposition in HIV-1-infected tissue samples [34,35]. GST constitutes the major intracellular antioxidant defense against reactive substances (RS) and oxidative stress. Moreover, the safe elimination of toxins *via* GST pathways was shown to protect cellular DNA against reactive oxygen species (ROS)-induced damage [36]. The up-regulation of GST in AIDS patients could have significant effect on HCV chronicity. Ran is a suppressor of Bcl-2-associated X protein (Bax), and a pro-apoptotic marker. HIV also induced the up-regulation of gelsolin that has been shown to inhibit HIV-induced T-cell apoptosis [20].

***HIV-1 down-regulated the expression of tropomyosin, profilin-1, and Rho-GDI.*** Tropomyosins forms coiled-coil dimers that assemble end-to-end along an actin filament [37] and provide an excellent example of spatial, temporal, and functional diversity [37,38]. Their observed down-regulation is consistent with the fact that the actin network is involved in HIV-1-induced host cell apoptosis [39]. Profilin-1 is an actin-binding protein thought to be a key regulator of actin polymerization in cells [40]. Profilin-1 has been shown to have an impact on HIV-infected macrophages [41]. Cytoskeletal proteins including profilin-1 have been shown to bind to HSP70 and to elicit resistance to simian immunodeficiency virus (SIV) infection of CD4^+^ T cells [42]. Previously, an impairment in the production of reactive oxygen intermediates has been demonstrated during HIV infection [43] and our finding of reduced Rho-GDI is in agreement with this observation. Rho-GDI has also been implicated in mediating HIV-infected cell migration through tight junctions [44] and was shown to be under-expressed in HIV-1-resistant women [45]. The mechanism by which these proteins are down-regulated remains to be investigated, but the up-regulation of mRNA (but not protein) for Rho-GDI and profilin-1 during HIV-1 infection could indicate impairment in protein translation induced by the virus. The possible interaction among these proteins during HIV-1 infection is represented in Figure 6A.

***HCV up-regulated tropomyosin, profilin, ER ATPase, HSP90-beta, thioredoxin, a protein highly similar to CBP2, DPYSL3, DPYSL4 and EIF3K.*** The up-regulation of a protein similar to CBP2 is in agreement with previous findings that HCV promotes expression of collagen [33]. Since CBP2 was also up-regulated in HIV-infected patients, it highlights a possible common molecular mechanism between these two viral infections. DPYSL3 and DPYSL4 are involved in semaphorin signaling and in subsequent rearrangement of the cytoskeleton [46,47]. EIF3K has been shown to be involved in apoptosis regulation by interacting with the subsequent liberation of caspase 3 into the cytosol [48]. After HCV infection, PBMC generate the over-expression of thioredoxin, inducing high levels of ROS that results in the down-regulation of GST (Figure 6B). In the cytoplasm, the liberated caspase 3 is known to cleave the Rho-GDI-Rac-GDP complex resulting in Rac activation, which is the first reaction for the cell apoptotic function [49].

***HCV down-regulated profilin-1 and Rho-GDI, as observed in HIV-1 infections.*** GST and GTPase Ran were also down-regulated in HCV-infected samples. The down-regulation of Rho-GDI may represent a pro-survival mechanism for both HIV-1 and HCV by reducing the activation of NADPH. It seems that during HCV infection, the over expression of thioredoxin triggers oxidative stress inducing high levels of ROS that result in the down-regulation of pro-apoptotic GST. The up-regulated caspase 3 cleaves the Rho-GDI-Rac-GDP complex resulting in Rac activation, which is the first step that leads to apoptosis [50]. The relevance of other down-regulated proteins with respect to HCV infection remains to be established. Figure 6B represent the possible contribution of observed proteins in HCV pathogenesis.

***HCV and HIV co-infection up-regulated CPB2 and the levels of expression of tropomyosin, profilin, and Rho-GDI as well as the pro-apoptotic proteins Annexin 5, NACHT, and TCP1.*** After the co-infection with HIV-1/HCV, oxidative stress could trigger the up-regulation of GST. In the cytoplasm, the up-regulated Rho-GDI is known to cause an anti-apoptotic signaling by inhibiting caspase 3 (Figure 6C).

***HCV and HIV co-infection down-regulated tropomyosin alpha-4 isoform 1, pyruvate kinase isozyme M1/M2, an ER ATPase, GST, EIF3K, and GTPase Ran*** of which the first three were down-regulated in HIV-1 but not in HCV-infected samples. Considering this, we postulate that their down-regulation is related to HIV-1 infection. Conversely, the down-regulation of GST and GTPase Ran might be due to the HCV infection. In addition, we observed that the protein, ER ATPase, a protein that is involved in ER-associated degradation pathway [51], was down-regulated in HIV-1 mono- and HIV-1/HCV co-infected samples but up-regulated during HCV mono-infection, indicating an inflammatory immune response in HCV infection and immune suppression in HIV-1 infection. More importantly, it appears that there is a divergence between immune activation and immune suppression during co-infection, which would explain the diversity of liver disease in the co-infected patients. Overall, interplay of identified proteins with relevance to HIV-1/HCV co-infection is presented in Figure 6C.

***The proteins unique to HIV-1/HCV co-infection included pro-inflammatory, apoptosis and immune response related proteins.*** For example, NLRP13 (NACHT, LRR, and PYD domains-containing protein 13) or NALPs are implicated in the activation of pro-inflammatory caspases (e.g., CASP1; MIM 147678) via their involvement in multi-protein complexes called inflammasomes [52]. Likewise, the NALP3 inflammasome is a crucial element in the adjuvant effect of aluminum and can direct a humoral adaptive immune response [53]. Annexin A5 (ANXA5) has been shown to be an important modulator of the immune response by interfering with the immunosuppressive effects of apoptotic and necrotic cells and certain viruses by preferentially binding phosphatidylserine with high affinity and inhibiting the uptake of these particles by macrophages [54], leading to defects in the clearance process that may cause chronic immune activity. Among the immune-related proteins identified only in the co-infected samples, TCP-1 (T-complex protein 1) and 60S acidic ribosomal protein P0 (RPL10) are worth mentioning. TCP-1, a molecular chaperone, assists the folding of proteins upon ATP hydrolysis and is known to play a role in the folding of actin and tubulin [55]. Activity-based proteome profiling of hepatoma cells using protease substrate probes identified TCP-1 as one of the few proteins differentially expressed during HCV replication [56], indicating the involvement of this chaperone in containing catalytic enzyme in HCV infection. Similarly, TCP-1 is differentially expressed in HCV polyprotein expressing cells [57]. P0 (60S acidic ribosomal protein) is a protein that is involved in host-virus interactions and has been shown to be up-regulated in HBV- and HCV-associated HCC and fibrosis progression [58,59]. These studies correlate with our findings and suggest that the co-infected PBMC protein profile is dominated by the proteins modulated primarily by HCV infection and that the similarity in the liver disease abnormalities in this patient population is due to the presence of HCV co-infection. The majority of the unique proteins identified were found in either HCV mono-infected or HIV-1/HCV co-infected samples. However, HSPA5, peptidyl-prolyl cis-trans isomerase A, and highly similar to phosphoglycerate kinase 1 proteins were not detected in infected PBMCs as compared to un-infected controls.

***Overall, our findings revealed that the majority of the differentially modulated proteins overlap between HCV mono- and HIV-1/HCV co-infected PBMCs***. It seems, that the liver disease progression is primarily HCV related and that HIV-1 co-infection may amplify the effects of stress and inflammation response prior to liver fibrosis, cirrhosis, and eventually carcinogenesis. In addition, the identified changes highlight protein signatures possibly involved in pro-inflammatory response and immune suppression that are highly implicated in progression of liver disease in chronic HCV infection and could potentially be used as drug targets. The specific pro and anti-apoptotic protein signatures revealed in this study could facilitate the understanding of protective immune-mediated mechanisms underlying HIV-1 and HCV co-infection. Accordingly, detailed analysis of the functional role of novel candidate molecular targets identified in this study would extend our understanding of the pathogenic effects of HIV, HCV and HIV/HCV co-infection and, in future, enable more specific and concurrent targeting of multiple key molecular pathways leading to better treatments.

## Abbreviations

AB: Ammonium bicarbonate; ACN: Acetonitrile; AFP: α-fetoprotein; AIDS: Acquired immunodeficiency Syndrome; CBP2: Collagen binding protein 2; GAPDH: Glyceraldehyde-3-phosphate dehydrogenase; GDP: Guanosine diphosphate; GTP: Guanosine triphosphate; HCV: Hepatitis C virus; HCC: Hepatocellular carcinoma; HIV-1: Human immunodeficiency virus type 1; HPLC: High-performance liquid chromatography; HSP90-beta: Heat shock protein 90 beta; IEF: Isoelectric focusing; IPI: International Protein Index; LC-MS/MS: Liquid chromatography-mass spectrometry and Mascot database; NADPH: Nicotinamide adenine dinucleotide phosphate; PBMC: Peripheral blood mononuclear cells; Rho-GDI-1: Rho GDP-dissociation inhibitor 1; ROS: Reactive oxygen species; STIP-1: Stress-induced phosphoprotein 1; SDS-PAGE: Sodium dodecyl sulfate-polyacrylamide gel electrophoresis.

## **Competing interests**

The authors declare that they have no competing interests.

## **Authors’ contributions**

NB, PJ and RP designed the study and experiments, NB, VS, JGA. C-d-R, MR, PS and ZN performed the experiments. AHT and LC provided reagents and samples. NB, PJ, RP and VS analyzed the results and wrote the manuscript. All authors read and approved the final manuscript.

## Supplementary Material

Additional file 1 **Table S1.** Differentially expressed proteins as analyzed by in-gel digestion and tandem mass spectrometry. ^1^MW = molecular weight in kDa; ^2^Exp = experimental; ^3^Theor = theoretical; ^4^pl = isoelectric point, number; ^5^P = peptide. *Protein levels of expression in ppm are depicted as shown in the scale at the bottom of the chart. Click here for file

Additional file 2 **Table S2.** Unique proteins identified by in-gel digestion and tandem mass spectrometry analyses. ^1^MW = molecular weight in kDA; ^2^Exp = experimental; ^3^Theor = theoretical; ^4^pl = isoelectric point, number; ^5^P = peptide. *Protein levels of expression in ppm are depicted as shown in the scale at the bottom of the chart. Click here for file

Additional file 3 **Table S3 and S4.** The additional data pertinent to the identification and characterization of proteins by mass spectrometry. Click here for file
