# Supplementary figures and images for: Unique and differential protein signatures within the mononuclear cells of HIV-1 and HCV mono-infected and co-infected patients
Source: Clin Proteomics. 2012 Sep 7;9(1):11. doi: 10.1186/1559-0275-9-11 (PMC3582525; doi:10.1186/1559-0275-9-11)

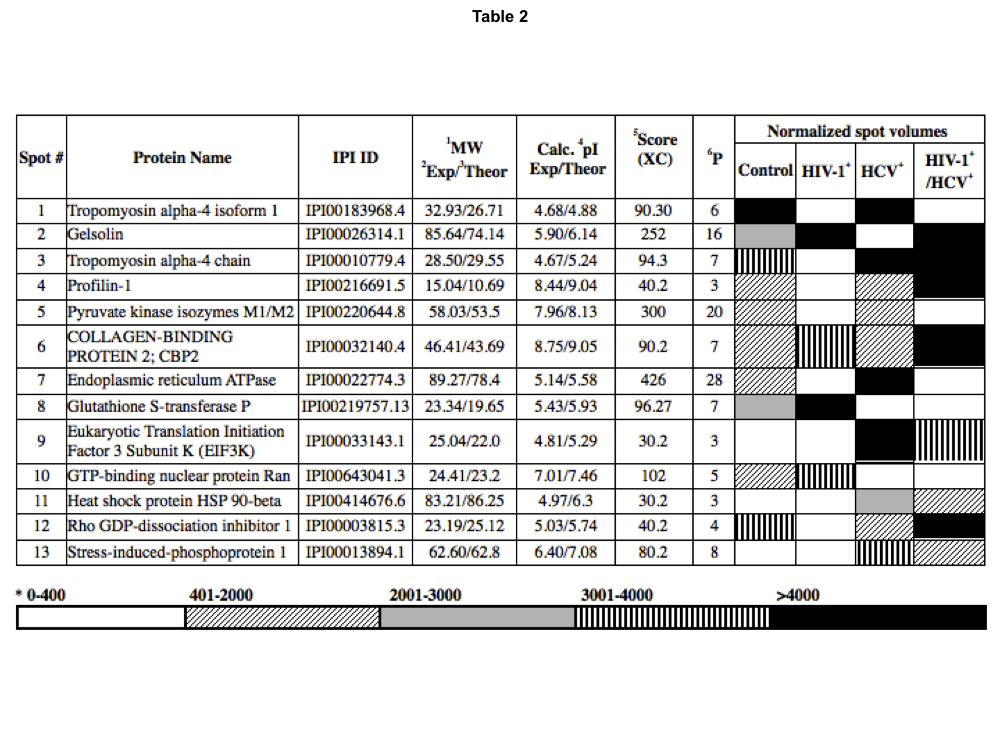

Supplement: Additional file 2 — Table S2. Unique proteins identified by in-gel digestion and tandem mass spectrometry analyses. 1MW = molecular weight in kDA; 2Exp = experimental; 3Theor = theoretical; 4pl = isoelectric point, number; 5P = peptide. *Protein levels of expression in ppm are depicted as shown in the scale at the bottom of the chart. [file 1559-0275-9-11-S2.tiff]

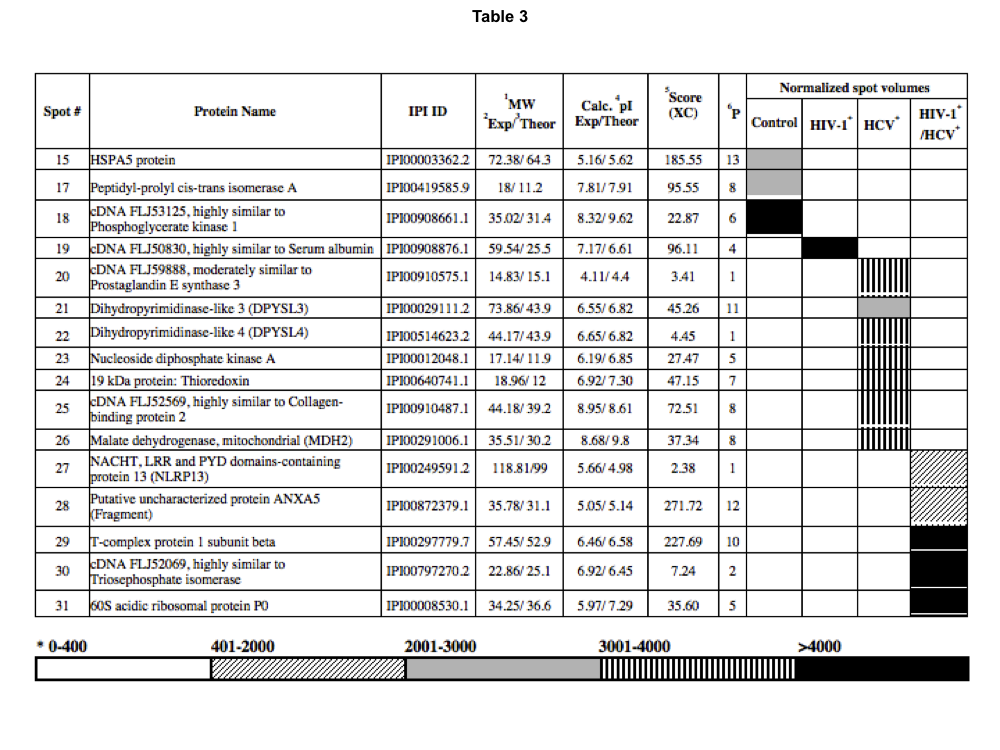

Supplement: Additional file 3 — Table S3 and S4. The additional data pertinent to the identification and characterization of proteins by mass spectrometry. [file 1559-0275-9-11-S3.tiff]
